# Emerging Wearable Biosensor Technologies for Stress Monitoring and Their Real-World Applications

**DOI:** 10.3390/bios12121097

**Published:** 2022-11-30

**Authors:** Ju-Yu Wu, Congo Tak-Shing Ching, Hui-Min David Wang, Lun-De Liao

**Affiliations:** 1 Institute of Biomedical Engineering and Nanomedicine, National Health Research Institutes, Zhunan Township, Miaoli County 35053, Taiwan; 2 Program in Tissue Engineering and Regenerative Medicine, National Chung Hsing University, South District, Taichung City 402, Taiwan; 3 Graduate Institute of Biomedical Engineering, National Chung Hsing University, South District, Taichung City 402, Taiwan; 4 Department of Electrical Engineering, National Chi Nan University, No. 1 University Road, Puli Township, Nantou County 545301, Taiwan

**Keywords:** wearables, biosensor, electrooculography (EOG) sensor, electroencephalography (EEG) sensor, sweat sensor, emotion evaluation, stress sensor

## Abstract

Wearable devices are being developed faster and applied more widely. Wearables have been used to monitor movement-related physiological indices, including heartbeat, movement, and other exercise metrics, for health purposes. People are also paying more attention to mental health issues, such as stress management. Wearable devices can be used to monitor emotional status and provide preliminary diagnoses and guided training functions. The nervous system responds to stress, which directly affects eye movements and sweat secretion. Therefore, the changes in brain potential, eye potential, and cortisol content in sweat could be used to interpret emotional changes, fatigue levels, and physiological and psychological stress. To better assess users, stress-sensing devices can be integrated with applications to improve cognitive function, attention, sports performance, learning ability, and stress release. These application-related wearables can be used in medical diagnosis and treatment, such as for attention-deficit hyperactivity disorder (ADHD), traumatic stress syndrome, and insomnia, thus facilitating precision medicine. However, many factors contribute to data errors and incorrect assessments, including the various wearable devices, sensor types, data reception methods, data processing accuracy and algorithms, application reliability and validity, and actual user actions. Therefore, in the future, medical platforms for wearable devices and applications should be developed, and product implementations should be evaluated clinically to confirm product accuracy and perform reliable research.

## 1. Introduction

### 1.1. The History of Wearable Biosensors

**Figure 1:**
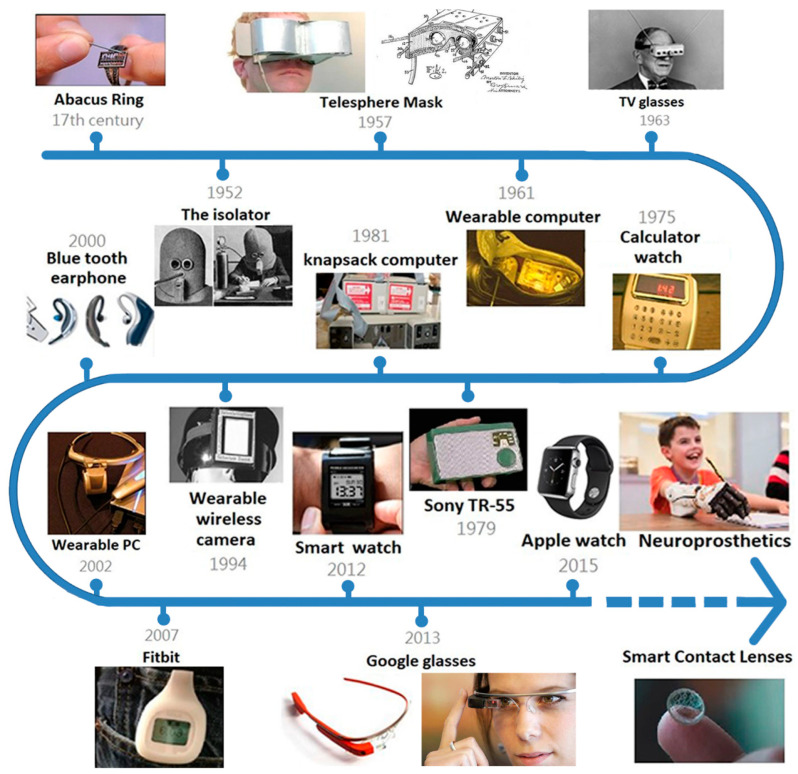
Evolution of wearable devices. The wisdom ring was developed in the 17th century [1]. In July 1925, Hugo Gernsback invented the isolation helmet [2]. In 1957, Morton Helig created the Telesphere Mask [3]. In June 1961, Edward Thorp and Claude Shannon invented the first portable computer [4]. In 1963, Hugo Gernsback designed a pair of TV glasses [5]. In 1975, the first computer watch was released. In the 1980s, the Walkman and knapsack computer were launched [1]. In 1994, Steve Mann developed head-mounted smart glasses [6]. In the 2000s, the iPod and Bluetooth were introduced. In 2002 and 2007, wearable PCs and the Fitbit were released. Since 2010, health watches, Google Glasses, Apple watches, and Oculus Rift headsets have been developed [1]. Since 2020, neuroprosthetics, smart contact lenses and other wearables have been investigated.

Wearable devices were developed earlier than is commonly believed. The timeline of milestones in the development of wearable devices is provided in Figure 1. The “wisdom ring” was invented in China as early as the 17th century. This abacus with seven rows of seven beads is only 1.2 cm long and 0.7 cm wide. This small size required the use of hairpins, which were typical hair decorations for women [1]. In the Western world, in July 1925, Hugo Gernsback invented the isolation helmet, which encloses the entire head and completely isolates the user from external noise to prevent distraction [2].

Currently, wearable devices usually refer to wearable electronic products, such as smart watches, smart bracelets, smart contact lenses, and smart tattoos. In 1955, Sony introduced the first transistor radio, the Sony TR-55, and other electronic wearables were developed soon after. In 1957, Morton Helig created the Telesphere Mask, a head-mounted display that shows 3D movies and provides stereo surround sound [3]. In June 1961, the first portable computer was invented by Edward Thorp and Claude Shannon, who were mathematics professors at MIT. This computer was only as large as a matchbox. Edward Thorp and Claude Shannon brought the device to a Las Vegas casino and tested it on a roulette wheel, where 44% of their predictions of the ball’s position on the wheel were correct [4]. In 1963, Hugo Gernsback invented a pair of glasses that combined two antennas, and TV channels could be selected through buttons on the front of the glasses [5]. In the 1970s, the functions of wearable devices became increasingly complex.

The first computer watch was released in 1975. The first head-mounted camera was also invented in the 1970s and was specifically designed for blind people. The camera was linked to a vest, and vibrations were generated on the vest to provide information about the surroundings. In the 1980s, the Walkman became a popular wearable music player. Wearable devices were used not only for amusement but also in health care. In 1987, the first digital hearing aid was released. Fitness trackers were also invented in this era. Sensors such as heart monitors, direction sensors and vibration detectors have been widely incorporated to increase the capabilities of wearable devices. Moreover, health awareness has increased, which led to the increasing use of wearable devices in health care in the 1990s [1].

In 1994, Canadian researcher Steve Mann developed head-mounted smart glasses and fixed them on his skull, requiring special tools for removal. Steve Mann also worked on improving this device. He invented EyeTab, which combines smart glasses with computers and the internet. Therefore, he is known as the father of wearable devices [6]. In the 1990s, Olivetti invented the portable Active Badge, which provided user locations via infrared signals. In the 2000s, the iPod and Bluetooth were developed, leading to progress in wearable devices, which became thinner, lighter, and wireless. Since 2010, the functions and sales of wearable devices have increased rapidly due to the popularity of the Internet of Things and wireless devices. Health watches, Google Glasses, Apple Watches, Oculus Rift headsets, etc., were developed in this era. In the 2020s, wearable devices have become closely related to our lives, with the possibility of movement information being evaluated and received instantly [1]. Wearable devices have shown significant research value and application potential in many fields, such as industry, medical care, the military, education, and entertainment. In 2018, Mary Meeker stated that wearables were the target of the third wave of industry cycles after personal computers, smartphones, and tablets [7].

With the increasing necessity of wearables, scientific research is developing rapidly. Specialized laboratories and research groups that focus on wearable smart devices have been established in many research institutions, such as Carnegie Mellon University, the Massachusetts Institute of Technology, the Korea Institute of Science and Technology, and the School of Engineering of the University of Tokyo. Innovative patents and technologies are being developed faster than ever before. The American Institute of Electrical and Electronics Engineers organized the Wearable IT Committee and wearable computing columns in several academic journals. The IEEE ISWC, an international scholarly conference on wearable smart devices, was held for the first time in 1997.

### 1.2. The Development Potential of Wearable Devices

**Figure 2:**
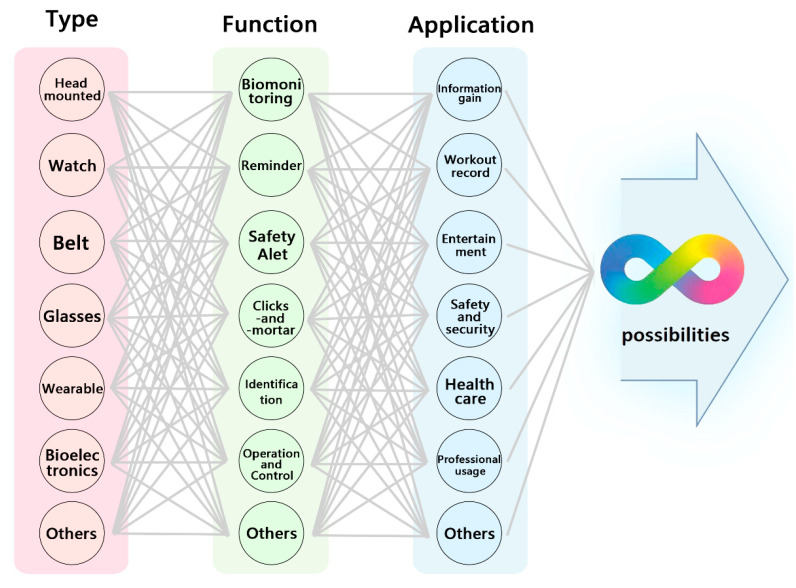
Application of wearable technology. Different shapes and materials are combined with various sensors and functions and adapted to specific applications, showing the potential of wearable devices. Different types of wearables, such as watches, bracelets, headphones, hats, shoes, clothes, necklaces, belts, patches, prosthetics, and glasses, have been developed. Wearables with functions related to biomonitoring, reminders, safety alerts, click and mortar retailing, identification, operation and control have been proposed. Finally, the functions can be applied for information gain, workout records, entertainment, safety and security, health care, and professional usage [8,9].

With the development of science and technology, physiological sensors and wearable devices with various forms have been proposed, including watches, bracelets, headphones, hats, shoes, clothes, necklaces, belts, patches, prosthetics, and glasses. Therefore, wearable devices have great development potential (Figure 2). Furthermore, with economic growth, the increasing prevalence of the internet, millennial population growth and the acceptance of intelligent technology, an increasing number of companies and research units are investing resources in the research, development and innovation of wearable devices and equipment.

Wearables offer significant economic benefits [8]. The COVID-19 pandemic and chronic diseases have changed lifestyles, which has accelerated the application of wearable technology in medical care and long-term measurement and monitoring. Wearable medical devices with wireless communication technology can easily collect physiological data according to the diagnostic needs, such as data related to cardiopulmonary function, exercise patterns, sweat content, tear content, tissue oxygen content, sleep, emotional state, cognitive function, and brain states after concussion. In addition, wearable devices can function as pulse oximeters, insulin pumps, and ECG monitors. After the medical data are collected and transmitted, the data can be uploaded to a platform and managed and interpreted by artificial intelligence. As a result, personal health care can be achieved remotely, which could decrease the number of hospital visits. These data can allow doctors and hospitals to assess the health status of individuals via telemedicine services, thereby reducing complications of chronic diseases. As a result, global digital healthcare market research is rapidly increasing. Due to the popularization of the 5G network, the improvement in sensor accuracy, and the creation of platforms with increasingly complete management and analysis capabilities, this market is expected to grow further [9].

### 1.3. Detection of Physical and Psychological Stress with Wearable Devices

Physical and psychological stress have attracted considerable attention because stress can affect multiple body and brain functions. Facial expressions, language, physiological states, biochemical values, and behavioral patterns change with depression, stress, anxiety, fatigue, sleep disturbance, lethargy, loss of appetite, and hyperactivity. Therefore, cortisol, prolactin, human growth hormone (hGH), adrenocorticotropic hormone (ACTH), and lactate in blood and saliva have been widely adopted to evaluate psychological stress. Biomarkers can be assessed invasively and noninvasively. Blood tests are the typical invasive method, and noninvasive methods include the detection of eye blinks [10], pupil dilation [11], electroencephalography (EEG) changes [12], and body fluid composition [13]. Through video monitoring, body language, gestures, and physical activity could be used to estimate users’ emotional states. Previous studies have confirmed that the visual stimuli in the International Affective Picture System (IAPS) could be used to arouse emotional responses. After five minutes of stimulation, which included groups of pleasant and unpleasant images, the subjects were assessed, including their body fluids. The researchers discovered that cortisol levels, EEG patterns, blink rates, and heart rhythm variability were all altered, which indicates that these data could potentially be utilized in stress detection systems [10].

When wearable devices are used for emotion detection, multiple factors must be comprehensively evaluated. Heart rate, heart rate variability, respiratory rate, blood pressure, EEG readings, electromyography (EMG) readings, electrooculography (EOG) readings, plethysmography (PPG) readings, galvanic skin response (GSR), skin temperature, etc. [14,15] could be used to assess emotions and stress level. These physiological changes can help assess and interpret the subject’s current external situation, executive motivation, and emotional development. Stress levels could be measured and monitored to protect the health of people working in stressful situations, such as doctors, members of the military, traffic controllers, and emergency service personnel (e.g., police, paramedics and firefighters) [16,17,18]. However, there are limitations regarding stress level recognition if only one detection method is used. This article introduces practical applications of wearable devices that detect brain waves, eye movements, and sweat components, the development of an emotion detection system, and the limitations of related research.

## 2. Electroencephalogram

### 2.1. What Is an EEG?

**Figure 3:**
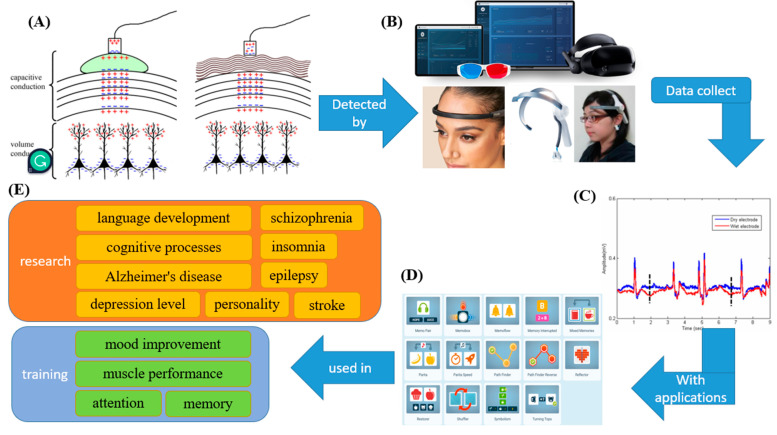
Wearable EEG detection device and application. (**A**) Neurons generate extracellular voltages, and the electrodes detect the sum of nearby positively and negatively charged areas. To amplify brain waves, gel or dry electrodes are essential [23]. (**B**) Wearables collect electrical signals [25]. (**C**) The data are collected and computed [24]. (**D**) Wearables can be used in various applications [26]. (**E**) The analyzed data could be used in research on depression, Alzheimer’s disease, epilepsy, schizophrenia, language development, cognitive processes, memory, attention, personality, stroke, insomnia, mood improvement, and muscle performance.

As the human brain operates, EEG signals arise from the activities of synchronized synapses among various populations of cortical neurons, which are pyramidal cells organized along cortical columns [19]. Near the dendrites, the excitation of a postsynaptic neuron generates an extracellular voltage that is more negative than other areas along the neuron, with regions of positive and negative charge separated by some distance. The sum of nearby positively and negatively charged areas is detected by the electrodes [20]. The difference in charge can be detected through the skin. However, the brain is not an organ of uniform size. Nerve cells have myelin sheaths, creating a physical barrier that ions cannot pass through. In addition, the different tissue densities in the brain can affect the propagation of electrical signals [21]. Moreover, the pia mater, dura mater, skull, and scalp are outside the brain and form a series of insulating layers that complicate electrical signal transmission. Therefore, a gel is required as a conductor to amplify the signals (Figure 3A) [22]. However, brain wave measurements are difficult to obtain because the eyes and muscles can generate electric fields. Furthermore, the skull and scalp barrier may attenuate the electrical signals. Therefore, how to correctly analyze EEG signals is also an essential issue [23,24].

German physiologist Hans Berger published the first human brainwave literature essay in 1929. Brainwaves were collected in the absence of stimulation. This means that the primary electrophysiological activity of the brain was measured [27]. The EEG signals of healthy people can be divided into α, β, θ and δ waves, according to the frequency and amplitude of the signals. The frequency of α waves is 8 to 13 times per second, and while these waves occur in all brain regions, they appear most often in the occipital region. In general, α waves show the basic rhythm of the healthy adult brain and are considered the best brain waves for learning and thinking. The frequency of β waves is 14 to 30 times per second, and these waves are more evident in the frontal, temporal and central regions. β waves are more widely observed during mental activity and emotional excitement, such as when a person’s eyes are opened and while thinking. In contrast, when the eyes are closed and at rest, β waves appear only in the frontal area. In general, the α wave is the primary brain rhythm. However, approximately 6% of people show evidence of β rhythms on EEG when mentally stable, indicating that β waves may be a fundamental wave. The frequency of θ waves is 4 to 7 times per second, and adults can often show evidence of this waveform when they are drowsy. In addition, θ waves are closely related to the limbic system, triggering deep memories and strengthening long-term memories. Therefore, the θ wave is “the gate to memory and learning”. The δ wave frequency is 0.5 to 3 times per second and often appears at the forehead. Healthy adults show evidence of this wave only when they are in a deep sleep. Therefore, θ and δ waves collectively refer to human rest states and are generally not registered in the awake state. Therefore, θ and δ waves are called slow waves [28].

### 2.2. How Is EEG Data Collected?

Gel is usually essential for detecting brain waves, as gel diminishes the impedance between the skin and the electrode surface. However, the gel hinders the use of brainwave detectors as wearable devices because gel leaves residues on the scalp, and electrode leakage could result in a short circuit between adjacent electrodes. Furthermore, gel dries out during prolonged use, resulting in a decrease in the EEG signal value [29]. Therefore, semidry or dry electrodes must be developed to replace gel-type wet electrodes [30]. Dry electrodes can be divided into three categories: contact electrodes, noncontact electrodes, and insulated electrodes. The main difference between noncontact and insulated electrodes is that the bottom of the insulated electrode is composed of insulating material. In contrast, noncontact electrodes are formed of metal and can be coupled with hair or clothing. Although the signal quality of contact electrodes is better, noncontact and insulated electrodes are easier to apply in wearable devices. However, dry electrodes still have limitations. Noise can increase quickly when the user moves [31]. Therefore, in 2011, Lin et al. developed a dry foam gel to replace wet electrodes. This dry electrode is composed of a conductive polymer foam made of polyurethane. Moreover, this dry electrode reduces the resistance between the skin and the electrode and can be used during movement. The signal qualities are better than those of the wet electrode during long-term EEG detection [24].

### 2.3. The Application of EEG in Measuring Physical Situations, Relieving Stress and Managing Emotions

When a specific physical or psychological event occurs during EEG recording, the voltage fluctuations in the EEG data can be measured and recorded, and the noise can be filtered. Then, the changes in brain potential can be calculated, and the event-related potential (ERP) can be determined (Figure 3C). The correspondence between EEG, ERP, and specific physical and psychological events could be used to assess the level of depression [32,33], Alzheimer’s disease [34,35], epilepsy [36,37], schizophrenia [38,39], language development [40,41], cognitive processes [42,43,44], memory [42,45], attention [45,46,47], personality [12], stroke [48,49], insomnia [50], mood improvement [12], and muscle performance [44,47,51,52] (Figure 3E).

Brain waves can not only indicate user situations but also improve external performance through brainwave training. For example, cognitive functions, such as working memory, attention, visual processing speed, and mental and emotional states, could be enhanced by deliberately changing eye movements, which can significantly boost optimal performance [52,53,54,55,56,57,58]. Surgeons can reduce surgery time by 26% and improve performance by training sensory motor rhythm-theta brain waves [52]. Therefore, many wearable devices that can perform EEG and EOG and detect other physiological states have been developed. Moreover, the data that are collected by wearables can be analyzed by associated applications. Then, the individual’s current situation could be determined, cognitive functions could be trained, and stress could be relieved. For example, Muse™ by InterAxon is an EEG detector that can be combined with other machinery and apps (e.g., Lowdown Focus, Opti Brain™ Version 3.12) for emotion management. Another application, NeuroTracker, was established 30 years ago as a research tool (Figure 3B) [59,60]. NeuroTracker was created as a training tool to improve cognitive functions, including working memory, visual processing speed, and attention [61,62]. NeuroTracker has been used to examine and teach muscle control to athletes [63,64], improve the cognitive function of concussion patients [65,66] and improve physical competence in older adults [67]. Many other wearables have been combined with applications, and this set of devices can be used to assess, estimate, establish and maintain concentration, message processing speed, memory, and problem-solving skills. These wearables include Fit Brains Trainer (Rosetta Stone, Arlington, VA, USA), Elevate Brain Training (Elevate, San Francisco, CA, USA), Lumosity (Lumos Labs, San Francisco, CA, USA) and (NeuroNation, Berlin, Germany). Furthermore, NeuroNation products can be reimbursed by German health insurance [26,68,69] (Figure 3D).

### 2.4. Emotion Detection with EEG

Emotional changes can be evaluated directly via EEG signals because emotions are related to voltage changes caused by the flow of ionic currents among neurons in the brain [70]. The parietal lobe is related to algesia, gustation, and critical thinking. The temporal lobe is responsible for hearing and memory. The occipital lobe manages vision-related tasks. The frontal lobe is primarily associated with sensation, critical thinking, speech, and movement [71]. The left hemisphere is more active during positive emotions, and the right hemisphere is more active during negative emotions [72,73] because the distribution of alpha waves is changed by different emotions [74]. This EEG signal asymmetry can be applied to emotion detection. Gonzalez et al. used the BioCNN hardware with a convolutional neural network to improve neural signal quality. The accuracy of emotion recognition was approximately 85%, which was higher than that obtained by other evaluation methods (approximately 77.57%) [75]. Moreover, the electrode type, the data collection algorithm, the willingness of users and the information processing methods could significantly impact the accuracy of emotion recognition [73,76,77].

### 2.5. Limitations of EEG Detection

Many factors affect EEG measurements. Electrode placement, electrode types, signal amplifier quality, signal processing methods, and user movements (eye movement, blinking, facial expression changes, etc.) can cause a considerable amount of noise, which can be difficult for EEG signals to handle. For EEG sensors to be used in wearables, the abovementioned problems must be addressed. Consequently, contact electrodes acquire data from only the prefrontal lobe, and other sensors, such as EOG, magnetoencephalography (MEG), body temperature and heartbeat sensors, are utilized to obtain additional information for comprehensive application judgment [78,79].

## 3. Eye Movement

### 3.1. In What Ways Do Eyes Move?

Eye movements can provide significant amounts of information. Human eyes move constantly, and some actions are unintentional or subconscious. For example, eyes enter a state of high-speed movement called saccades when we read, where the eyes move rapidly from word to word. When we enter a room, our eyes make broad sweeps of the surroundings. Eyes also make small, unconscious movements to counteract head shaking and provide stable vision when we walk. During sleep, eyes move from side to side during the rapid eye movement stage. Eye movements have been shown to reveal thinking processes [80,81]. Moreover, pupil dilation is associated with uncertainty in decision-making [81,82], and eye movements can facilitate memory retrieval [83,84,85]. The following methods of measuring eye movement are discussed: eye tracking and EOG.

### 3.2. How Can Eye Movement Data Be Collected?

#### 3.2.1. Eye Tracking

Eye movement detection data have been widely used in research, such as work related to cognitive load recognition, reading comprehension, presentation design, distraction and attention guidance, and some human–computer interfaces (HCIs) [80]. Eye trackers are a standard system for detecting different eye movements and are composed of small cameras that can be installed on tables, shelves or in unique caps with no eye contact. Eye position data can be recorded to track the pupil position, fixation time, fixation direction, fixation repetitions, saccade length, and pupil diameter. This type of device is a video-based eye tracker [86,87].

However, there are some difficulties in measuring and utilizing eye movement data. Physiological studies have found a speed difference between the eyes during eye saccades. Eye movements to the nasal side are slower, while those to the outside world are faster. This slight gap, known as the fixational disparity, is positively related to the saccade distance. This slight gap is most likely due to the change in the distance between two letters while reading; the eyes converge after staring, which indicates that this fixational disparity could be adjusted by various circumstances [88]. Therefore, sitting posture, binocular correction, and head and body movement significantly impact eye movement detection. The physiology of eye movements and machine correction are technical issues in measuring eye movements [89,90].

#### 3.2.2. EOG

**Figure 4:**
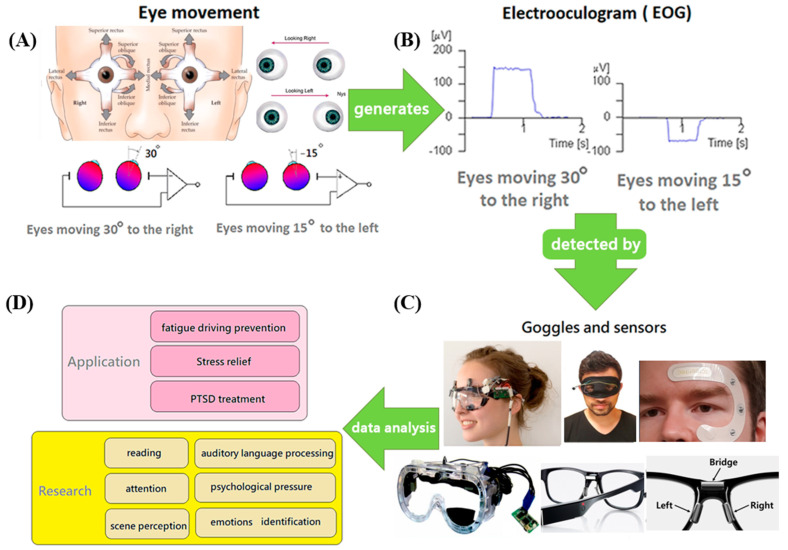
Wearable EOG detection device and application. (**A**) Six muscles control the fine-motor movements of each eye [93]. (**B**) Electrooculography (EOG) signals are positive when the eyes move toward the right (horizontally). In contrast, EOG signals are negative when the eye moves to the left [91,92]. (**C**) Several goggles and sensors have been developed to detect EOG signals [10,94,95,96]. (**D**) After data analysis, the wearables for detecting EOG could be applied in fatigued driving prevention [97], stress relief and PTSD treatment. Moreover, EOG wearables can be used as research equipment to explore the mechanisms of reading, attention, scene perception, auditory language processing, psychological pressure and emotional identification.

Some limitations in eye movement detection are due to environmental and user issues. Therefore, eye potential measurement is a solution for movement tracking. Eye potential refers to the potential difference of several tens of mV generated between the cornea and retina of the eye (Figure 4A). In general, the human cornea side is positively charged, and the retina side is negatively charged; thus, a potential difference can be recognized (Figure 4B). To measure this potential difference, electrodes can be placed on the skin around the eyes, and the possible changes were found to result from eye movements and blinking. The sensor could detect and measure the potential difference, which could be used to investigate the line of sight, mental state, and fatigue level [91,92]. Recently, EOG sensors have been adopted in medical applications, which are associated with embedded user interfaces (UIs) for improved usage. These sensors can be used in drowsiness detection [92].

Compared to EEG, EOG shows a better signal, higher amplitude, better signal-to-noise ratio, and easier recording conditions [98]. However, EOG signal collection suffers from similar problems as EEG signal collection. For example, the sensing electrodes require the use of gel between the skin and the electrodes to enhance the response, or the sensors need to be wired. Regardless of the electrode type, it can be inconvenient or uncomfortable for users [97,99]. Therefore, in the development of new wearables for EOG measurement, two to three dry electrodes were embedded in eyeglass frames and nose pads [10,94]. In another study, some electrodes were made of silver and fixed in the appropriate location by a polyurethane membrane to collect data [100] (Figure 4C). Regardless of the electrode placement method, the wearing comfort, convenience, and data collection accuracy must be addressed by developers.

### 3.3. Different Glasses-Type Wearables for Fatigue Detection and Human-Computer Interfaces

Eye movements provide a considerable amount of information that can be applied in neuroscience, psychology, industrial engineering, human factors engineering, marketing/advertising, computer science, etc. [101]. Psychology research has worked to obtain information on reading [101,102,103], scene perception [104,105,106], problem solving [106,107], auditory language processing [108,109], attention [110], emotion recognition and psychological stress [17,111], PTSD treatment and emotional stress relief [112]. Therefore, glasses-type wearable devices for EOG or eye movement detection could be applied to investigate fatigued driving, identify emotional changes, and treat PTSD and in applications related to reading, attention, image perception, problem-solving, and auditory-language processing (Figure 4D).

In eye-assisted selection and entry (EASE), the time of eye fixation is used as an indicator, and maneuvering a cursor on a screen usually requires between 78.81 and 131.45 ms of sustained focus [113]. In 2006, EASE studies showed that only 81% of the subject choices were correctly classified by eye trackers. This human-machine interaction using eye movements is highly dependent on the design of the interface. “KIBITZER” is a wearable device that was developed in 2010 that can detect the line of sight of users and the current scene through an eye tracker on the head, and two camera lenses increase the accuracy of eye movement data. The eye movement data are transmitted to a computer, allowing the user to receive text or voice explanations to better understand the environment. However, this system has some shortcomings. When the environmental light is too bright, the pupil size and gaze-tracking functions can be disturbed. In contrast, when the environment is dark and sufficient artificial light sources are not provided, the system cannot detect the environment [114].

Due to the limitations of eye trackers, different applications related to EOG signals have been developed in human-machine interface research, such as eye disease recognition [115], virtual computer keyboard and mouse control [116,117], wheelchair control [118], computer games [119], machine control [120], and simplified Chinese eye writing systems [121]. However, the accuracy of the obtained data is limited if the data are collected only by eye trackers or EOG measurements. Multimodal eye movement signal recording methods that incorporate EEG, EMG, and eye trackers can significantly improve human-machine interface system performance [122,123].

In 2021, a device that detects human eye movements via video-based methods, EOG signals and infrared oculography was developed. Infrared oculography uses the reflection of near-infrared light on the cornea to determine the eye position and can be used in dark environments. In this system, hardware and software are adopted through the cooperation of video systems, dual Purkinje images and optical eye-tracking systems to improve image processing. With this system, eye movement detection accuracy has dramatically improved. The highest accuracy of approximately 96% was achieved with upward eye movements, while looking to the right resulted in the lowest accuracy of approximately 87.67%. Almost 100% of invalid information, such as blinking and wide-ranging glances, was ignored. This human–computer interaction system for detecting eye movements can distinguish ten eye movements (i.e., up, down, left, right, far left, far right, upper left, lower left, upper right, and lower right). Moreover, this system provides an eye–dial interface that can enable patients with amyotrophic lateral sclerosis (ALS), brainstem stroke, brain or spinal cord injury, muscular dystrophy, cerebral palsy, or multiple sclerosis to express their intentions and smoothly communicate with others [95]. However, this system has some drawbacks. Infrared sensors are expensive to manufacture, and long-term infrared exposure may cause eye discomfort.

“JINS MEME” is a glasses-type wearable device that was released by the Japanese glasses manufacturer JIN in May 2014. This device is equipped with an eye potential sensing function, and through eye movement detection and blinking, the intention and physical state of users can be inferred in real time. Gaze movement can be embedded in smartphone and in-vehicle device applications. Users can turn pages and select icons by looking at the smartphone screen. Through the continuous acquisition and analysis of gaze and blink information, the system can detect user fatigue and drowsiness because the eyes make unique movements, such as irregular or rapid blinking, when people want to sleep or are stressed [10]. Thus, JINS MEME could help prevent drowsy driving [124,125]. In 2015, another study combined EEG and oxyhemoglobin concentration detection to develop a fatigue detection system that could more accurately determine state changes during fatigued driving [126,127,128].

There are advantages and disadvantages to using EOG and EEG in human–computer interaction techniques. EOG signals may extend to the entire scalp when human eyes are rotated, which may cause interference between EOGs and EEGs. EOG detection in human–computer interactions requires considerable physical strength for some patients who cannot use their eye muscles well. In contrast, if only EEG data are desired, EOGs are a primary source of interference. Brainwave fragments disturbed by EOGs are usually deleted, which is called the rejection method and may lead to data loss and bias and failure to observe eye movement-related effects. Therefore, EOG signals could be measured independently and subtracted from EEG data using various regression methods to provide data without human interference. Many methods of removing EOG signals from EEGs have limitations [129,130,131]. Although EEG detection consumes less physical power, EEG signals have higher noise and lower accuracy than EOG signals. Therefore, the combination of EOGs and EEGs provides better results in human-computer interactions [132,133,134]. Thus, many wearable devices have combined EEGs and EOGs. When detecting fatigue levels and sleep behaviors, EEG signals are often added for collective judgment to obtain accurate interpretations [135,136].

### 3.4. Emotion Detection by EEGs

User interest, visual search processes, and information processing have been analyzed using eye movements in previous studies. Moreover, the blink frequency, fixation rate, maximum fixation duration, total fixation dispersion, maximum fixation dispersion, saccade rate, average saccade duration, saccade delay, average saccade amplitude, and EOG signals can reflect emotional performance [137]. Pupil diameter is the most commonly used feature for emotion recognition. The pupil diameter increases during positive emotions. Therefore, pupil size is considered to be a reliable indicator of positive emotion [138]. Anwar et al. used the pupil position and size captured by eye tracking devices and the fixation duration and proposed a facial expression recognition and eye gaze estimation system. Happiness, anger, sadness, neutral emotions, surprise, disgust, and fear could be recognized by the system, and the rate of correct emotion discrimination was approximately 90% [139]. Raudonis et al. divided emotions into four types, neutral, disgusted, funny and interested, and used eye movement speed, pupil size and pupil position for emotion recognition. The average recognition accuracy was approximately 90%, and the highest precision was obtained when classifying “interest” [140]. However, detecting emotions via eye movements requires multiple variables to increase the recognition accuracy to 85%. If only EOG data are used to detect emotions, the recognition rate for four emotions is approximately 88.3% [141] or 77.11% [142]. If only the pupil size is used to determine emotion, the accuracy rates are only 58.9% [143] and 59% [144]. Therefore, it is necessary to use a multimodal method in emotional judgment to improve the data accuracy and diversify the identifiable emotions.

### 3.5. Limitations of Eye Movement Detection

The movement of human eyes is affected not only by the physiological and psychological conditions of the individual but also by the light intensity of the environment and the type of electrodes. The accuracy of signal processing affects the interpretation of the data. Therefore, multimodal collaborative judgment is essential for addressing the limitations of eye movement detection [100,133,145,146]. Furthermore, the various electrodes, sensors, signal receivers and algorithms developed for wearable devices have made eye movement detection more popular.

## 4. Sweat Detection

### 4.1. Sweat Components

Most biomarkers are present in the blood, and sweat is transported to the surface of the skin through dermal ducts. Therefore, biomarker concentrations in sweat could correspond to those in the blood and can be considerable indicators of human health [147]. The ions, metabolites, acids, hormones, small proteins, and peptides secreted in sweat can be detected to identify various physiological states [148,149]. Additionally, sweat glands are found in almost every part of the skin and can easily be accessed without using needles or other invasive devices [147]. As a result, sweat detection has become the most popular method in wearable biomonitoring research, more so than the detection of tears, interstitial fluid (ISF), breath, saliva, or wound exudate [150].

Sweat is secreted by sweat glands, and this process is regulated by the autonomic nervous system. The excretion and evaporation of sweat helps to carry away a large amount of heat, thereby cooling the individual, and the sweat components not only remove waste but also moisturize the skin, soften keratin, cause the epidermis to acidify and inhibit the breeding of bacteria. Sweat is mainly composed of water (99%), and the remaining elements include electrolytes (sodium, potassium, magnesium, and calcium), lactic acid, urea, ammonia, protein, carbonate, cortisol, and various neuropeptides and cytokines [151]. The concentration of sweat compounds varies among individuals due to the differences in sweat gland activity and secretion rate. The average person sweats only approximately 600–700 mm^3^ per day [152].

### 4.2. How Can the Components of Sweat Be Detected?

Sweat can continuously and noninvasively provide abundant biomarker measurements of ions, drugs, metabolites, and biomolecules, including K^+^, Na^+^, Ca^2+^, Cl^−^, lactate, glucose, ammonia, ethanol, urea, cortisol, and various neuropeptides and cytokines. Different methods for analyzing sweat have been developed. For example, potentiometry and square wave stripping voltammetry can be used to detect ions in sweat [153,154,155], and chronoamperometry can be used to detect drugs [147,156,157,158], glucose [155,159,160], and metabolites, such as lactate [161] and peptides [162], in sweat. In addition to bioelectronics-related methods used to examine sweat components, specific bioassays that combine metallic materials with antibodies have been developed to detect small concentrations of chemicals in sweat. For example, cortisol in sweat can be measured by zinc oxide nanosheets, which combine with cortisol antibodies and are very sensitive (they can detect concentrations ranging from 1 to 200 ng/mL in sweat). Cortisol can reflect psychological or physiological stress [163,164,165]. Muscle activity has been predicted by K+ [153] and lactate concentrations in sweat. The quantities of these two compounds in sweat are the same as those in blood [154]. Thus, K+ and lactate could be used to evaluate the physical exertion and exercise intensity of users [105,106]. In addition, the glucose concentration in sweat can be used to continuously monitor blood glucose [155].

In addition to body function judgment and analysis, sweat detection can conveniently be used to detect physiological and psychological stress. Sweat is normally secreted to regulate body temperature. Nevertheless, when a person’s anxiety level increases, the autonomic nervous system produces a series of physiological responses to adapt to stress, such as increased heart rate, pupil dilation and sweat secretion that occurs over the entire skin, especially on a person’s palms, soles, and underarms; this response is usually unrelated to ambient temperature [15,156] However, sweating increases and decreases during mental relaxation and sleep [157]. The amount of lactic acid and urea in sweat increases significantly when individual stress increases [17]. Previous studies have shown that fearful people have disgusted facial expressions [158], increased alertness [159,160], and stronger smelling sweat. However, various stresses can affect hormone secretion, such as corticotropin-releasing factor (CRF), ACTH, mineralocorticoids, insulin, and hGH [161,162]. In 2016, a combination of sensors and antibodies was developed, in which gold nanoparticles and cortisol antigens were coated on the surface of the sensor. When cortisol bound to the surface, the gold nanoparticles turned red. This technique is a stable and competitive method that can be used to develop color-changing wearables to detect user stress levels [163].

### 4.3. The Application of Sweat Detection to Determine Body Movement and Emotion

The components in sweat are complex and easy to detect, and various sweat component detection sensors have been developed. In 2021, sweat-detecting colorimetric ion-selective photoelectrodes were weaved into the fabrics of innovative sportswear to detect pH, Na+ and K+ concentrations in sweat to assess the physical activity level of the wearer [155]. Moreover, two smartwatches called Cortisol Apta-Watch (CATCH) and CortiWatch can detect the quantities of cortisol in sweat to reflect physical and mental stress. With minimal errors, cortisol concentrations in sweat can be revealed by zeta potential, Fourier transform infrared spectroscopy (FTIR), and electrochemical measurements. Therefore, people could be informed about their stress levels, and stress levels could easily be monitored, allowing doctors to provide more straightforward diagnoses and treatments [150,164].

### 4.4. Limitations of Sweat Detection

Wearable devices that use sweat detection to assess physical or psychological health offer great benefits. The components and concentrations in sweat can represent the chemicals in the blood. Thus, diabetes, kidney function, exercise intensity, cystic fibrosis and alcohol concentration could be monitored, and physiological changes could be uncovered [150]. However, many difficulties must be overcome. The composition ratios in sweat differ from those in plasma. Thus, converting sweat concentrations into plasma concentrations is the most critical issue that must be solved. Moreover, the quantities and chemical amounts in sweat differ among individuals, and microelectrodes cause the skin to sweat. Whether the composition of the induced sweat is the same as that of actual sweat needs to be confirmed. The activity patterns of individuals also affect sweat secretion [150,166]. Thus, sweat composition analysis may be sufficiently accurate to estimate physical and psychological states.

## 5. Summary and Outlook

Wearable devices for physiological detection have been adopted in various applications, such as monitoring personal health, assessing and controlling psychophysiological states, evaluating stress, preventing brain lesions, and controlling robots, robotic arms or devices such as wheelchairs, by detecting eye movements, brain waves and sweat [69,167,168]. This technology has become a necessity for some people. In addition to monitoring physiological values, wearable devices have been increasingly used to detect emotions, reduce stress and facilitate cognitive training. Therefore, an increasing number of research institutions are focusing on EEG, EOG, and cortisol applications to improve data accuracy in emotional detection. Many technical difficulties must be overcome in the development of new technology. The activity patterns of users should not affect the accuracy of the sensor, and the sensitivity of the sensor should be adjustable. Moreover, the efficacy of wearable sensors must be scientifically confirmed. These problems need to be addressed. Commercial products that are composed of wearables and biosensors have little evidence to support the benefits that they claim, and their practical value is controversial [169,170]. It is crucial to determine whether the physiological data collected by these devices are accurate and if it is medically ethical to collect these data. In the future, the increasing use of wearables in health and sports performance could benefit researchers in collecting data [171]. The cooperation between biosensors and applications could support the development of precision medicine, generate substantial financial profits and improve the quality of life of users. Therefore, health and performance technology companies should collaborate with researchers to confirm the efficacy and scientific value of their products so that biosensors can be used in everyday life [171].

## Data Availability

The data that support the findings of this study are available on request from the corresponding author.
